# Do associations between suicide ideation and its correlates (substance use, anxiety, and depression) differ according to victimization type among youth? A Smart platform study

**DOI:** 10.1016/j.pmedr.2022.101944

**Published:** 2022-08-10

**Authors:** Nour Hammami, Tarun Reddy Katapally

**Affiliations:** a Trent University Durham, 55 Thornton Road South, Oshawa, ON L1J 5Y1, Canada; b DEPtH Laboratory, Faculty of Health Sciences, Western University, 1151 Richmond St, London, ON N6A 5B9, Canada; c Epidemiology and Biostatistics, Schulich School of Medicine and Dentistry, Western University, 3rd Floor, Western Centre for Public Health and Family Medicine, 1465 Richmond Street, London, ON N6G 2M1, Canada

**Keywords:** Youth health, Citizen science, Mental health, Suicide, Bullying, Cannabis use, Substance use

## Abstract

The issues associated with mental health, substance misuse, and suicide ideation are complex and sensitive among youth. We sought to investigate the role that subjective health, internalizing and externalizing risk factors play in the association between victimization and suicide ideation among youth in Canada via used a custom-built digital epidemiological smartphone application (Smart Platform) on their personal smartphones.

A sample of 818 youth citizen scientists in Saskatchewan, Canada downloaded the app to provide information on victimization, subjective health, internalizing problems (symptoms of stress, anxiety, and depression), externalizing behaviours (cannabis use, alcohol, smoking), and suicide ideation. Binary regression models were used to estimate associations and controlled for gender, age, perpetration, and ethnicity.

From our sample, 23% of youth reported suicide ideation (i.e., thoughts) in the past year. Three types of victimization (cyberbullied, made fun or teased, or bullied via being left out) are associated with a two-times higher risk of suicide ideation. Although certain risk factors (anxiety, poor subjective health, and cannabis use) were associated with higher suicide ideation risk, they did not moderate the association between victimization and suicide ideation. Symptoms of depression were found to be protective against suicide ideation.

Suicide ideation is high among this sample of youth in Canada. Certain types of victimization, internalizing and externalizing risk factors, and poor subjective health are associated with a higher risk of suicide ideation. However, our findings confirm that the pathway from victimization to suicide ideation is complex and is potentially moderated by factors other than the ones explored here.

## Highlights

• 23% of the youth reported suicide ideation in the past year.
• Suicide ideation was higher among those victimized by bullying.
• Suicide ideation was also higher with reported anxiety, or poor subjective health.
• Poor health did not play a role between victimization and suicide ideation.

## 1 Introduction

Suicide is the second leading cause of death globally and among Canadians aged 15 to 24 years (Campisi et al., 2020, Findlay, 2017). Recent evidence from a nationally representative sample indicates that among individuals aged 15 to 24 years in Canada, 14 % report suicidal thoughts (suicide ideation) in their lifetime and 6 % in the past 12 months (Findlay, 2017). These numbers are lower than global estimates which indicate that 18 % of adolescents reported suicide ideation in their lifetime and 14.2 % reported suicide ideation in the past 12 months (Lim et al., 2019). Suicide ideation is associated with several risk factors (Islam et al., 2021), one of which is peer victimization (Ford et al., 2017). Peer victimization (i.e., bullying) is a form of youth violence consisting of unwanted aggressive behaviour, by other youth(s), that is repeated, and involves a perceived power imbalance (Centers for Disease Control and Prevention, 2021).

Youth who have been victimized or who have perpetrated victimisation are significantly more likely to present with suicide ideation, relative to youth not involved in victimization, as reported by several systematic reviews and *meta*-analyses (Katsaras et al., 2018, Kowalski et al., 2014, van Geel et al., 2014). An explanation is that victimization is a stressor on youth (McDougall and Vaillancourt, 2015) and this strain leads to internalizing problems and behaviours (e.g., self-injury) as the General Strain Theory posits (Agnew, 2001, Hay and Meldrum, 2010). Literature supports that youth who were victimized reported lower subjective well-being (Bradshaw et al., 2017, Chen and Elklit, 2018), more internalizing problems (mainly depression) (Álvarez-García et al., 2015, Cook et al., 2010, Hong et al., 2015), and more externalizing behaviours (such as substance misuse) (Álvarez-García et al., 2015, Cook et al., 2010, van Noorden et al., 2015) than youth not involved with victimization.

Research also suggests that internalizing problems and externalizing behaviours have a moderating role in the association between victimization and suicide ideation. Depression has been reported to have a role (Brunstein klomek et al., 2007, Klomek et al., 2009) (Bauman et al., 2013); while substance misuse has been reported to partially mediate and intensify the relationship among youth (Litwiller and Brausch, 2013). Other than depression, few studies investigated the role of other internalizing problems in the association between victimization and suicide. A study on Emirati youth investigated the role of internalizing problems in the association between victimization and suicide (Itani et al., 2018). Both feeling lonely and worried were significantly associated with higher suicide risk when controlling for victimization; however, the role these internalizing problems played via an interaction term was not investigated (Itani et al., 2018). More recent research assessed for the mediating role of internalizing problems in the association between victimization and suicidal thoughts among African American youth in the U.S. The authors reported that depression, low self-esteem, and hopelessness have an indirect mediating role in the association between victimization and suicidal thoughts (Lee et al., 2021). Given the scarcity of the available research, it is important to examine how moderating factors amplify the association between victimization and suicide (Hong et al., 2015).

There is research to support that certain types of victimization have a distinct and different association with suicide ideation. A *meta*-analysis by van Geel et al., (2014) reported that the association is stronger between cybervictimization and suicide ideation than between traditional victimization and suicide ideation. This association may also be different by victimization status as a systematic review and *meta*-analyses found that only cyberbullied youth (not perpetrators) were more likely to have presented with suicide ideation (Katsaras et al., 2018).

A comprehensive investigation that considers both internalizing problems and externalizing behaviours in assessing the relationship between victimization (traditional and cyber) and suicide ideation is needed. This study builds on existing literature by investigating whether subjective health, internalizing problems (symptoms of stress, anxiety, and depression), externalizing behaviours (cannabis use, alcohol, smoking), play a role in the association between victimization (traditional and cyber) and suicide ideation among youth aged 13–18 years (Katapally, 2020a, Katapally, 2020b).

## 2 Methods

### 2.1 Design

As part of a digital epidemiological and citizen science platform (Smart Platform), (Katapally, 2020a, Katapally, 2020b) a quasi-experimental study called Smart Youth was conducted immediately after the legalization of cannabis in Canada (see Fig. 1.a.) (Government of Canada, 2020). 818 youth (aged 13–18 years) in 5 out of 12 high schools in the provincial capital city of Regina, Saskatchewan, Canada, were engaged as citizen scientists via their own Smartphones. All youth used a custom-built digital epidemiological Smartphone application (app), specifically adapted for the study, which operates on both Android and iOS platforms. Ethics approval was obtained from the Research Ethics Boards of Universities of Regina and Saskatchewan through a synchronized review protocol (REB # 2017–29).

**Fig. 1a:**
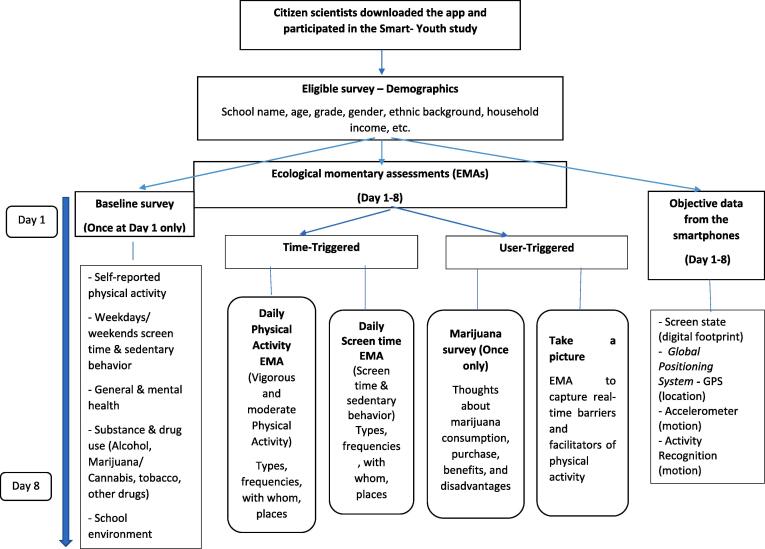
Smart youth survey deployment.

### 2.2 Recruitment

Twelve high schools in the city of Regina were approached to participate in the study, out of which 5 schools agreed to participate. One week before recruitment, schools shared implied informed consent forms with youth’s caregivers via email. Caregivers were given the option to reach out to the research team to opt their children out of the study. In close coordination with each school, the Smart Platform research team conducted separate recruitment presentations to students in each grade (grades 8–12). After class presentations, youth who decided to participate in the study downloaded the custom-built app onto their own Smartphones and provided informed consent via the app. The overall participation rate of youth was over 80 % across all schools, thus resulting in a representative sample.

As soon as youth joined the study via the app, eligibility, and baseline surveys were triggered, which were completed in the presence of our team on day 1 of the study. The Smart Youth Survey utilized a combination of validated questionnaires to capture a complex set of health behaviours and outcomes, including physical activity, victimization, sleep, suicide ideation, mental health (including anxiety and depression symptoms), and substance misuse.

### 2.3 Participants

Participants in the Smart Platform are “citizen scientists” as they engage with the researchers at all stages of the research process. Citizen scientists informed the design, research questions and outcome measures of this study. Our citizen engagement is governed by a Citizen Scientist Advisory Council, consisting of citizens of varied age cohorts (13–18, 18–25, 25–50, greater than50 years), genders, ethnicities, and socioeconomic status from Saskatchewan, Canada. The Advisory Council informs conceptualization, implementation, and evaluation of Smart Platform studies.

## 3 Measures

### 3.1 Suicide ideation

Suicide ideation was measured via the question “During the past 12 months, did you ever seriously consider attempting suicide?” and there were 2 response options: Yes or No (see Fig. 1.b), in line with the Youth Risk Behavior Survey Questionnaire (Centers for Disease Control and Prevention (CDC), 2017).

**Fig. 1b:**
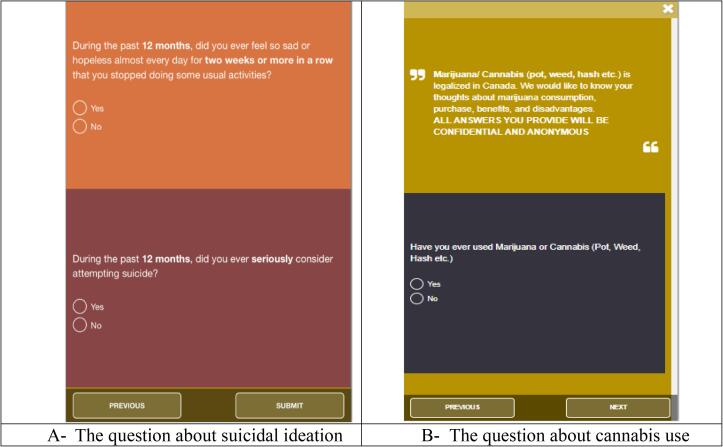
Screenshots of suicidal ideation and Cannabis questions.

### 3.2 Victimization

#### 3.2.1 Victimized youth

Victimization was measured by asking “How many times did these things happen in the last 30 days” with the categories “other students shoved or hit you”, “other students left you out of things on purpose”, “other students called you mean names”, “other students made fun of or teased you in a hurtful way”, “other students told lies or spread false rumours about you”, “other students used social media, Facebook, texting, emailing, etc. to tell lies about you, embarrass you, and threaten you”. For our analyses, we grouped the responses to result in the following 2 categories: never or yes in the past week.

#### 3.2.2 Perpetrators of victimization

As for perpetration, it was measured with the survey question “I encouraged students to tease, push, or shove other students” and “I joined in when students told lies about other students”. There were 4 response options for all questions: never, about once a week, 2 or 3 times a week, daily or almost daily. For our analyses, we grouped the responses to result in the following 2 categories: never or yes in the past week.

### 3.3 Subjective health

Self-rated health was measured with the question “In general, would you say your health is?” with 5 response options: Very good, good, fair, bad, very bad. For this analysis, we grouped the categories very good, good, and fair to form one category and the second category grouped bad and very bad.

Self-rated mental health (SRMH) was measured with the question “In general, would you say your mental health is?” with 6 response options: Poor, fair, good, very good, excellent, I don’t know. For this analysis, we grouped the categories fair, good, very good, and excellent to form one category and the second category was poor. Those who replied with I don’t know were considered missing (n = 18).

### 3.4 Internalizing behaviours

Symptoms of stress were measured via the question “Thinking about the amount of stress in your life, would you say that most days are…” and there were 5 response options: Not at all stressful, not very stressful, a bit stressful, very stressful, extremely stressful. For this analysis, we grouped the categories Not at all stressful, not very stressful to form one category and the second category grouped a bit stressful, very stressful, extremely stressful.

Screening positively for anxiety was determined via the Generalized Anxiety Disorder (GAD-2)’s two questions on anxiety: “How often over the last 2 weeks were you bothered by feeling nervous, anxious, or on edge” and “How often over the last 2 weeks were you bothered by not being able to stop or control worrying?” with the response options: not at all, several days, more than half the days, or nearly every day (Löwe et al., 2010). The response options were summed with the lowest (zero) indicating no anxiety symptoms and highest (six) indicating nearly every day experiencing symptoms listed in the GAD-2’s questions. As per the guidelines, youth with a GAD-2 score of 3 or higher were classified as screening positive for generalized anxiety disorder, otherwise they did not (Kroenke et al., 2007).

Symptoms of depressions were measured via the question “During the last 12 months, did you ever feel so sad or hopeless almost every day for two weeks or more in a row that you stopped doing some usual activities?” with the response options yes or no, in line with the Youth Risk Behavior Survey Questionnaire (Centers for Disease Control and Prevention (CDC), 2017).

### 3.5 Externalizing behaviours

Cannabis ever use was measured with the question “Have you ever used Marijuana or Cannabis (Pot, Weed, Hash etc.)” (see Fig. 1.b.). Alcohol ever use was measured with the question “Have you ever consumed alcohol in your life?”. Tobacco ever use was measured with the question “Have you ever used any tobacco related products?”. All these questions had 2 response options: Yes or No.

### 3.6 Ethnicity, age, and gender (independent variables)

Ethnicity was measured with the question “How would you describe your ethnic background? *Select all that apply.*” and there were 13 response options: First Nations, Dene, Cree, Metis, Inuit, African, Asian, Canadian, Caribbean/West Indian, Eastern European, European, South Asian, Other (please specify). In the analyses, ethnicity’s response options were grouped to result in the follows categories: Indigenous, Canadian, Other Ethnicity(ies).

Gender was measured with the question “What is your gender?” and there were 5 response options: Male, Female, Transgender, Other (please specify), Prefer not to disclose. Due to the low cell count in the latter 3 categories, in this analysis, gender was categorized into 3 categories: female, male, transgender/other (please specify)/prefer not to disclose.

Age was measured with the open-ended question “How old are you? (age in years)”.

### 3.7 Statistical analyses

Smart youth who had more than 75 % of responses missing in their questionnaire were excluded (n = 381); as such, the analyses represent findings from the remaining 437 Smart youth. Summary characteristics are presented as frequencies. Binary regression models were used for the multivariate analyses which assessed the associations between victimization types, internalizing, externalizing factors, subjective health, and suicide ideation. All the models controlled for ethnicity, gender, school, and age. Bully perpetration was not found to be associated with suicide ideation (dependent variable); as such, it was used as a control in all models. Relative risk ratios and 95 % confidence intervals are reported, and significance was set at p < 0.05. All analyses were conducted in Stata 15.0 (StataCorp., 2015).

## 4 Results

### 4.1 Characteristics of the sample

Table 1 shows characteristics of the sample across sociodemographic factors, victimization behaviours, internalizing behaviours, subjective health, and externalizing behaviours arranged according to whether youth had suicide thoughts in the past year or not. The table also indicates whether there is a significant difference across the respective factor and suicide ideation via Pearson χ^2^. Youth who had suicidal thoughts in the past year were 22.8 % of the sample.

**Table 1:** Summary characteristics (in percent) of a sample of youth in Canada and by whether or not they had suicidal thoughts in the past year.

		Suicide ideation			
	Total	No	Yes	Pearson χ^2^	P-value
**Gender**					
Female (n = 225)	56.1	53	67		
Male (n = 153)	38.2	42.8	21.6		
Transgender / Other / Prefer not to disclose (n = 23)	5.7	4.2	11.4		
Total (n = 401)	100	100	100	16.7	<0.0001
**Ethnicity**					
Indigenous (n = 20)	5	4.8	5.7		
Canadian (n = 162)	40.5	41.3	37.5		
Other (n = 218)	54.5	53.8	56.8		
Total (n = 400)	100	100	100	0.47	0.792
**Physically victimized (shove or hit)**					
Never (n = 334)	81.1	84.9	68.1		
Yes, in past week (n = 78)	18.9	15.1	31.9		
Total (n = 412)	100	100	100	13.4	<0.0001
**Verbally victimized (name-calling)**					
Never (n = 295)	71.8	76.7	55.3		
Yes, in past week (n = 116)	28.2	23.3	44.7		
Total (n = 411)	100	100	100	16.3	<0.0001
**Cyberbullied**					
Never (n = 361)	88.5	93	73.4		
Yes, in past week (n = 47)	11.5	7	26.6		
Total (n = 408)	100	100	100	27.2	<0.0001
**Relationally victimized (made fun or teased)**					
Never (n = 310)	75.6	81.7	54.8		
Yes, in past week (n = 100)	24.4	18.3	45.2		
Total (n = 410)	100	100	100	28.1	<0.0001
**Relationally victimized (false rumors spread)**					
Never (n = 317)	77.7	81.8	63.8		
Yes, in past week (n = 91)	22.3	18.2	36.2		
Total (n = 408)	100	100	100	13.5	<0.0001
**Relationally victimized (left out of things on purpose)**					
Never (n = 298)	72.5	78.9	51.1		
Yes, in past week (n = 113)	27.5	21.1	48.9		
Total (n = 411)	100	100	100	28.1	<0.0001
**Perpetrator of physical victimization (shove or hit)**					
Never (n = 380)	92.5	93.1	90.3		
Yes, in past week (n = 31)	7.5	6.9	9.7		
Total (n = 411)	100	100	100	0.78	0.375
**Perpetrator of relational victimization (false rumors spread)**					
Never (n = 385)	93.9	94.6	91.4		
Yes, in past week (n = 25)	6.1	5.4	8.6		
Total (n = 410)	100	100	100	1.32	0.251
**Stress**					
No (n = 83)	20	23.4	8.4		
Yes (n = 332)	80	76.6	91.6		
Total (n = 415)	100	100	100	10.3	0.001
**Anxiety in last 2 weeks**					
No (n = 284)	68.3	78.1	34.7		
Yes (n = 132)	31.7	21.7	65.3		
Total (n = 415)	100	100	100	63.5	<0.0001
**Depressed last 12 months**					
No (n = 173)	41.7	29.4	83.2		
Yes (n = 242)	58.3	70.6	16.8		
Total (n = 415)	100	100	100	87.1	<0.0001
**Self-rated mental health**					
Fair/ Good/ Very Good/ Excellent (n = 330)	82.5	91.9	51.1		
Poor (n = 70)	17.5	8.1	48.9		
Total (n = 400)	100	100	100	81.7	<0.0001
**Self-rated health**					
Very Good/ Good/ Fair (n = 376)	92.8	96.8	79.8		
Bad/Very bad (n = 29)	7.2	3.2	20.2		
Total (n = 405)	100	100	100	31.4	<0.0001
**Ever used alcohol**					
No (n = 250)	61.3	57.6	73.4		
Yes (n = 158)	38.7	42.4	26.6		
Total (n = 408)	100	100	100	7.6	0.006
**Ever used cannabis**					
No (n = 308)	74.8	80.4	55.8		
Yes (n = 104)	25.2	19.6	44.2		
Total (n = 412)	100	100	100	23.5	<0.0001
**Ever used tobacco**					
No (n = 89)	21.5	16.4	38.9		
Yes (n = 324)	78.5	83.6	61.1		
Total (n = 413)	100	100	100	22.1	<0.0001

The sample consisted of 56.1 % females and 38.2 % males, while 5.7 % youth reported being transgender, other, or preferred not to disclose their gender identity. Only 5 % youth identified as Indigenous, while 40.5 % identified as Canadian, and 54.5 % reported belonging to different ethnicities.

In the past week, youth reported being physically victimized (18.9 %), verbally victimized (28.2 %), cyberbullied (11.5 %), relationally bullied: made fun of or teased (24.4 %), false rumours spread about them (22.3 %), left out of things on purpose (27.5 %). All these victimized behaviours were statistically different across suicide ideation.

More youth reported suicidal thoughts with the respective internalizing behaviours, relative to those experiencing the respective internalizing behaviour but without suicidal thoughts (statistically significant difference, seen in Table 1). For example, of those who reported being stressed, 91.6 % also had suicidal thoughts, relative to those stressed but without suicidal thoughts (76.6 %). Similar observations are made for: screening positively for anxiety, poor subjective health, and cannabis use.

### 4.2 **Regression** analyses

Table 2 shows risk ratios and 95 % confidence intervals for the associations between suicide ideation and victimization among each of: number of victimizations (model 1), all individual victimizations (model 2), and among significant victimizations identified in model 2 (model 3). The number of victimization types is associated with suicide ideation, suggesting a dose–response relationship. Three victimization behaviours (cyberbullied, made fun or teased, or bullied via being left out) are associated with 2.94 (95 % C.I. = 1.18 – 7.31), 2.42 (95 % C.I. = 1.10–5.35), and 2.24 (95 % C.I. = 1.12–4.448) higher risk of suicide ideation, respectively (model 2). The associations were slightly attenuated but remained significant when the non-significant victimization behaviours were removed from the model (results of model 3).

**Table 2:** Risk ratios (and 95% Confidence intervals) showing associations between suicide ideation and victimization among each of: number of victimizations (model 1), all individual victimizations (model 2), and among significant victimizations identified in model 2 (model 3) among a sample of youth in Canada.

	Suicide ideation		
	Model 1	Model 2	Model 3
**Number of victimization types** (Ref. = 0)			
1	2.57*		
	(1.20–5.50)		
2	4.75**		
	(1.94–11.62)		
3	6.81***		
	(2.42–19.15)		
4	7.79***		
	(2.78–21.78)		
5	9.64***		
	(3.16–29.41)		
6	5.87**		
	(1.73–19.88)		
**Physically victimized (shove or hit)** (Ref. = No)			
Yes		1.12	
		(0.48–2.63)	
**Verbally victimized (name-calling)** (Ref. = No)			
Yes		0.90	
		(0.39–2.09)	
**Cyberbullied** (Ref. = No)			
Yes		2.94*	2.70*
		(1.18 – 7.31)	(1.16 – 6.31)
**Relationally bullied (made fun or teased)** (Ref. = No)			
Yes		2.42*	2.23*
		(1.10–5.35)	(1.13–4.42)
**Relationally bullied (false rumors spread)** (Ref. = No)			
Yes		0.80	
		(0.35–1.80)	
**Relationally bullied (left out of things on purpose)** (Ref. = No)			
Yes		2.24*	2.15*
		(1.12–4.48)	(1.12–4.11)

*** p < 0.001, ** p < 0.01, * p < 0.05; 95 % Confidence intervals in parentheses Models controlled for perpetrating victimization, gender, ethnicity, school, and age.

Table 3 shows the associations between internalizing behaviours (symptoms of stress, anxiety, and depression), externalizing behaviours (alcohol use, cannabis, tobacco), subjective health, and victimization. Victimization is associated with higher risk of certain internalizing factors. Specifically, relational victimization was associated with a higher risk of feeling stressed (R.R. = 3.24, 95 % C.I. = 1.27–8.23, results of Model 1); physical victimization was associated with a higher risk of anxiety (R.R. = 2.51, 95 % C.I. = 1.14–5.55, Model 2) and cannabis use (R.R. = 2.90, 95 % C.I. = 1.21–6.91, Model 5), and verbal victimization and cybervictimization were associated with a higher risk of poorer self-rated mental health (R.R. = 3.98, 95 % C.I. = 1.58–10.05 and R.R. = 2.57, 95 % C.I. = 0.89–7.37, Model 7), respectively. Two types of victimizations were associated with a lower risk of depression and lower risk of reporting poor self-rated mental health, respectively cybervictimization (R.R. = 0.31, 95 % C.I. = 0.12–0.79, Model 3) and false rumours spread (R.R. = 0.20, 95 % C.I. = 0.068–0.58, Model 7).

**Table 3:** Risk ratios and 95% confidence intervals for the associations between internalizing factors (symptoms of stress, anxiety, depression), externalizing factors (alcohol use, cannabis, tobacco), poor subjective health, and victimization.

	Internalizing problems			Externalizing behaviours			Subjective health	
	Stress	Anxiety	Depression	Alcohol	Cannabis	Tobacco	SRMH	SRH
	Model 1	Model 2	Model 3	Model 4	Model 5	Model 6	Model 7	Model 8
**Physically victimized (shove or hit)** (Ref. = No)								
Yes	0.84	2.51*	1.22	0.52	2.90*	0.23**	1.56	0.73
	(0.34–2.06)	(1.14–5.55)	(0.54–2.72)	(0.24–1.11)	(1.21–6.91)	(0.089–0.62)	(0.59–4.08)	(0.17–3.20)
**Verbally victimized (name-calling)** (Ref. = No)								
Yes	0.66	1.45	0.56	0.79	0.83	0.94	3.98**	1.38
	(0.28–1.55)	(0.68–3.07)	(0.27–1.15)	(0.39–1.58)	(0.32–2.13)	(0.32–2.79)	(1.58–10.05)	(0.31–6.10)
**Cyberbullied** (Ref. = No)								
Yes	0.70	0.94	0.31*	0.73	0.96	0.37	2.57	2.81
	(0.24–2.05)	(0.38–2.29)	(0.12–0.79)	(0.30–1.76)	(0.36–2.60)	(0.13–1.06)	(0.89–7.37)	(0.65–12.05)
**Relationally bullied (made fun or teased)** (Ref. = No)								
Yes	1.52	1.69	0.53	1.04	0.83	1.60	2.12	1.47
	(0.58–4.00)	(0.80–3.56)	(0.25–1.11)	(0.52–2.10)	(0.31–2.21)	(0.50–5.06)	(0.80–5.61)	(0.35–6.27)
**Relationally bullied (false rumors spread)** (Ref. = No)								
Yes	0.86	1.00	1.65	0.87	1.96	0.53	0.20**	0.67
	(0.35–2.14)	(0.48–2.11)	(0.79–3.45)	(0.43–1.77)	(0.84–4.58)	(0.20–1.40)	(0.068–0.58)	(0.16–2.70)
**Relationally bullied (left out of things on purpose)** (Ref. = No)								
Yes	3.24*	1.52	0.57	1.31	1.09	1.06	1.37	2.31
	(1.27–8.23)	(0.79–2.94)	(0.30–1.07)	(0.71–2.43)	(0.49–2.44)	(0.40–2.78)	(0.59–3.18)	(0.72–7.39)

***p < 0.001, ** p < 0.01, * p < 0.05; 95 % Confidence intervals in parentheses.

Models controlled for perpetrating victimization, gender, ethnicity, school, and age.

Table 4 shows risk ratios and 95 % confidence intervals for the associations between suicide ideation and internalizing problems (Model 1), subjective health (Model 2), and externalizing behaviours (Model 3). Among the internalizing factors, anxiety was associated with a 2.85 higher risk of suicide ideation (95 % C.I. = 1.53–5.30, Model 1) while symptoms of depression were associated with a lower risk of suicide ideation (R.R. = 0.13, 95 % C.I. = 0.064–0.25). As for subjective health, poor SRMH and bad or very bad SRH were associated with 11.21 (95 % C.I. = 5.58–22.52, Model 2) and 4.83 (95 % C.I. = 1.70–13.75, Model 2) times higher risk of suicide ideation, respectively. Among substance misuse, only cannabis use is associated with a higher risk of suicide ideation (R.R. = 3.62, 95 % C.I. = 1.67–7.83, Model 3).

**Table 4:** Risk ratios and 95% confidence intervals for the associations between suicide ideation and internalizing factors (Model 1), subjective health (Model 2), and externalizing factors (Model 3).

	Suicide ideation		
	Model 1	Model 2	Model 3
**Symptoms of stress** (Ref. = No)			
Yes	1.29		
	(0.48–3.47)		
**Screened positively for anxiety** (Ref. = No)			
Yes	2.85**		
	(1.53–5.30)		
**Symptoms of depression** (Ref. = No)			
Yes	0.13***		
	(0.064–0.25)		
**Self-rated mental health** (Ref. = Fair/ Good/ Very Good/ Excellent)			
Poor		11.21***	
		(5.58 – 22.52)	
**Self-rated health** (Ref. = Very Good/ Good/ Fair)			
Bad/Very bad		4.83**	
		(1.70–13.75)	
**Ever used alcohol** (Ref. = No)			
Yes			0.79
			(0.42–1.51)
**Ever used cannabis** (Ref. = No)			
Yes			3.62**
			(1.67–7.83)
**Ever used tobacco** (Ref. = No)			
Yes			0.47
			(0.20–1.07)

*** p < 0.001, ** p < 0.01, * p < 0.05; 95 % Confidence intervals in parentheses.

Models controlled for perpetrating victimization, gender, ethnicity, school, and age.

Table 5 builds on previous findings by showing estimates for suicide ideation while including in the model the victimization behaviour and the factor it has an association with from Table 3. All internalizing factors and victimization behaviours show significantly higher risk of suicide ideation except for depression. Thus, when controlling for internalizing factors, victimization behaviours are associated with suicide ideation; the reverse is also true, when controlling for victimization behaviours, internalizing factors are associated with suicide ideation. The same is applicable to ever used cannabis and SRMH.

**Table 5:** Suicide & internalizing and victimization (significant associations tested only).

	Suicide ideation				
	Model 1	Model 2	Model 3	Model 4	Model 5
**Symptoms of stress** (Ref. = No)					
Yes	2.48*				
	(1.02–6.05)				
**Screened positively for anxiety** (Ref. = No)					
Yes		5.18***			
		(2.90–9.22)			
**Symptoms of depression** (Ref. = No)					
Yes			0.102***		
			(0.051–0.19)		
**Self-rated mental health** (Ref. = Fair/ Good/ Very Good/ Excellent)					
Poor				15.54***	
				(7.34 – 32.94)	
**Ever used cannabis** (Ref. = No)					5.05***
Yes					(2.68–9.52)
**Physically victimized (shove or hit)** (Ref. = No)					
Yes		1.72			2.16*
		(0.85–3.48)			(1.08–4.34)
**Relationally bullied (left out of things on purpose)** (Ref. = No)					
Yes	3.37***				
	(1.92–5.90)				
**Relationally bullied (made fun or teased)** (Ref. = No)					
Yes					
**Cyberbullied** (Ref. = No)					
Yes			3.41**	3.31*	
			(1.48–7.87)	(1.22 – 8.97)	
**Verbally bullied (name-calling)** (Ref. = No)					
Yes				0.83	
				(0.38–1.80)	
**Relational victimization (false rumors spread)** (Ref. = No)					
Yes				2.34*	
				(1.02–5.38)	

*** p < 0.001, ** p < 0.01, * p < 0.05; 95 % Confidence intervals in parentheses.

Models controlled for perpetrating victimization, gender, ethnicity, school, and age.

### 4.3 Interaction terms

Building on results from Table 5, we developed additional models that tested whether there was a moderating effect that internalizing problems, cannabis use, and SRMH played in the association between victimization behaviours and suicide attempts, respectively. Internalizing problems, cannabis use, and SRMH did not moderate the association between victimization and suicide ideation (results not shown since statistical significance was not evident).

## 5 Discussion

This study is the first comprehensive investigation that ethically engaged youth (13–18 years) as citizen scientists (Katapally, 2020a, Katapally, 2020b) via their own smartphones to understand how internalizing problems and externalizing behaviours moderate the relationship between victimization (traditional and cyber) and suicide ideation. The primary aim was to highlight the complexity of issues that impact youth mental health, while providing them an opportunity to securely report these issues using their mobile devices.

The first major finding among our sample of youth, was that the suicide ideation was almost 5 times the national average at 23 % compared to the 6 % reported at a national level in 2014 (Statistics Canada, 2014). This is a statistic that cannot be ignored and should be explored further by engaging with youth in real-time using their mobile devices. As stigma is a major barrier to seeking mental health supports (Moskalenko et al., 2020), it is critical to confirm whether youth have been consistently under-reporting mental health issues; and perhaps more importantly, whether we can use advanced digital citizen science methods (Katapally, 2020b, Katapally, 2020b, Katapally et al., 2018) to engage youth ethically using their own mobile devices to minimize barriers to reporting.

The prevalence of mood disorders among our sample was also higher than Canadian national averages for: generalized anxiety disorders (31.7 % versus 2.4 %), depressive episodes (58.3 % versus 7.1 %), and fair or poor subjective mental health (17.5 % versus 8.1 %) (Statistics Canada, 2014). Only cannabis ever use was less than the national average (25.3 % versus 44.8 %) (Statistics Canada, 2014). Although our findings refer to mental health symptoms not diagnoses, these differences highlight the importance of early screening for mental health symptoms as their prevalence is high. Additionally, these findings may be showing a pattern of either under-reporting using traditional survey methods or over-reporting among our sample; either way, it is necessary to conduct further studies using digital tools to confirm or refute evidence generated in our study.

Our findings show that suicide ideation has associations with behavioural risk factors (i.e., cannabis use), internalized risk factors (i.e., anxiety), poor self-rated mental health, and social risk factors (i.e., victimization by peers), in line with previous research (Guo et al., 2021, Katsaras et al., 2018, Kowalski et al., 2014, Nock et al., 2013, van Geel et al., 2014). Following the General Strain Theory, other risk factors should be investigated in the association between victimization and suicide ideation (Agnew, 2001, Hay and Meldrum, 2010). While an association pathway linking these factors was not identified in our analyses, these findings do not discount that suicide behaviour prevention should be multifaceted (Miller and Coffey, 2021). Other risk factors that can be explored include victimization’s association with personality development. Self-mastery is a personality trait that was recently found to mediate the association between cyberbullying victimization and symptoms of depression and social anxiety (Wang, 2021). Understanding the role that victimization plays in personality development among youth is an area of exploration which will highlight pathways of associations that have not been yet explored.

A clinical diagnosis of depression is reportedly the strongest predictor of suicidal behaviour (Zelazny et al., 2021). However, in our sample and in other samples, symptoms of depression were found to be protective against suicide ideation. Reportedly, about half of youth in Canada who had depression or suicidal thoughts sought professional help (Findlay, 2017, Statistics Canada, 2018). Although our study did not address clinical diagnoses, it is worthy to note that psychotic illnesses such as borderline personality disorder have been found to be associated with both victimization and suicide ideation (Choo *et al.*, 2014). This emphasizes the importance of investigating clinical and psychotic illnesses in future studies that address victimization and suicide ideation. Other research confirms that mental health services are an effective protective mechanism against suicidal behaviours, as are family cohesion and strong interpersonal relationships (Abraham and Sher, 2021, King et al., 2018).

There are mixed findings as to whether school and community-based interventions are effective (Kutcher et al., 2017, Siu,, 2019), an area of public health that could benefit from digital health interventions (Lattie et al., 2019). Co-designing digital health interventions with youth is critical for their success (Bergin et al., 2020), and to co-create digital health interventions, it is imperative to engage with youth ethically, an approach that digital citizen science can enable (Katapally, 2020a, Katapally, 2020b, Katapally, 2020b, Katapally, 2020b).

Our study shows that relative to the national Canadian average, disadvantaged communities, particularly rural and remote Indigenous communities, are carrying a heavier burden of mood disorders and suicidal behaviours, while facing inequities in terms of lower access to mental health services (Boksa et al., 2015). This gap in access can be minimized by incorporating digital health interventions, particularly through mobile apps. Mobile apps have been found to be effective in improving mental health outcomes among youth in remote communities (Tighe et al., 2017).

Since smartphone-based digital health interventions can detect within person changes for individuals and can recommend appropriate mental health services (Sreejith and Menon, 2019, Wang et al., 2018), they could provide a much-needed service to address existing gaps in current health systems. Although there is research to support that screen time is associated with poorer health outcomes among youth, there is also research to support that digital health interventions can minimize the negative health effects that they target (Grekin et al., 2019, Katapally and Chu, 2019, Song et al., 2019, Yang and Van Stee, 2019). Youth in rural communities tend to use mental health help-lines less than youth in urban areas (Thompson et al., 2018). As such, offering remote and online options for mental health promotion and services will reduce barriers to access due to location, and this should be coupled with the promotion of these services in remote communities (Thompson et al., 2018).

### 5.1 Strengths and limitations

Citizen scientist perspectives obtained through smartphone reporting are prone to recall and social desirability bias. Youth may have under- or over-reported symptoms and behaviours. However, we believe that smartphone engagement provides anonymity that reduces misreporting (World Health Organization, 2019).

The exposures and outcomes that we examined were cross-sectional, raising issues of temporality and reverse causality. Our study does not consider youth who may have dropped out of school, and these data should be collected in future studies. Although we engaged youth to obtain comprehensive information, residual confounders may not have been accounted for in our analysis (e.g., information about seeking professional mental health services, stigma of seeking professional mental health services) – another area of focus for future data collection.

The primary strength of our study is that it provides insight into both big data collection and policy interventions using digital citizen science. With over 6 billion smartphone subscriptions globally (Statista, 2021), the ability to engage as citizen scientists changes the landscape of population health research (Katapally, 2019, Katapally, 2020b). In fact, all youth who agreed to participate in our study owned smartphones with data plans. As part of the digital citizen science-based Smart Platform (Katapally, 2020a, Katapally, 2020b), to address potential Internet inequity (Katapally, 2019, Katapally, 2020b), we work with schools to ensure that all youth and who participate as citizen scientists receive access to mobile phones and data plans.

## 6 Conclusion

Suicide ideation has many risk factors, which need to be captured appropriately before developing a multifaceted approach for prevention. Digital citizen science could potentially enable this approach by enabling ethical engagement with youth as well as deployment of real-time digital health interventions using mobile devices.

## CRediT authorship contribution statement

**Nour Hammami:** Conceptualization, Methodology, Data curation, Formal analysis, Writing – original draft. **Tarun Reddy Katapally:** Conceptualization, Methodology, Validation, Writing – review & editing, Supervision, Funding acquisition.

## Declaration of Competing Interest

The authors declare that they have no known competing financial interests or personal relationships that could have appeared to influence the work reported in this paper.

## Data Availability

The data that has been used is confidential.
